# Persistent *Streptococcus pyogenes* infection of the forearm following blunt trauma

**DOI:** 10.1080/23320885.2020.1858715

**Published:** 2020-12-12

**Authors:** Erin M. Cravez, Adam Y. Nasreddine, Andrea Halim

**Affiliations:** Department of Orthopaedics and Rehabilitation, Yale University School of Medicine, New Haven, CT, USA

**Keywords:** Necrotizing fasciitis, soft tissue infection, toxic shock

## Abstract

Necrotizing soft tissue infections are aggressive manifestations of *Streptococcus pyogenes*, often described after minor skin trauma. However, a subset of infections may present without cutaneous findings. We report a case of toxic shock syndrome and recalcitrant streptococcal infection of the forearm in a healthy teenager following blunt trauma.

## Introduction

Group A Streptococcus (*Streptococcus pyogenes*) is a virulent organism implicated in a wide spectrum of diseases, from mild pharyngitis to severe necrotizing soft tissue infections (NSTI) and streptococcal toxic shock syndrome (STSS). A review of the global burden of *Streptococcal pyogenes* from 2005 estimates that group A streptococcal diseases account for more than 600 million infections annually and approximately 500,000 deaths from severe disease and sequelae [1]. Although STSS only accounts for 4% of cases, it is subject to a high mortality rate (38%) [2,3].

Reaching a diagnosis of STSS can be challenging as patients typically present with a myriad of non-specific symptoms and laboratory findings. A recent case series identified the most common presenting symptoms of pediatric patients to include fever (89%), gastrointestinal complaints (67%), respiratory distress (56%), cellulitis (33%) and a macular rash (32%) [3]. The most common etiology for systemic STSS are soft tissue infections such as necrotizing fasciitis, myositis, or cellulitis, accounting for up to 80% of cases [4]. It is therefore critical that soft tissue infections with or without necrotizing features are promptly identified and addressed with appropriate antibiotic therapy and early surgical intervention. Although NSTI is classically described as being associated with a minor breach in the skin or mucous membrane, up to 50% of cases are ‘cryptogenic infections’ without an identifiable skin lesion [4]. Since cutaneous findings are less apparent, diagnosis is more often delayed and mortality can exceed 78% [4–6].

We herein present a case report of a healthy 14-year-old boy who developed STSS related to a persistent non-necrotizing soft tissue infection after blunt trauma without any identifiable skin break, requiring repeated debridement. We believe the unusually tenacious nature of this infection highlights the need to maintain high clinical suspicion and frequent surveillance of patients with suspected group A strep infection.

## Case summary

A fourteen-year-old previously healthy lacrosse player sustained a blunt checking injury to his left proximal forearm during a sports camp without disruption of the overlying skin. He had mild pain focal to the site but was able to continue play. That evening he developed nausea, vomiting, and insidious periumbilical abdominal pain. The next day, he developed a fever to 102 F as well as increased left arm swelling, prompting his family to seek care at an urgent clinic. Imaging of the forearm was unrevealing and given his fever and primary complaint of abdominal pain, he was felt to have a viral gastroenteritis. Two days after injury, his abdominal pain and vomiting progressively worsened and he developed a cough, sore throat, and new-onset hematuria, resulting in hospital admission.

**Figure 1.**
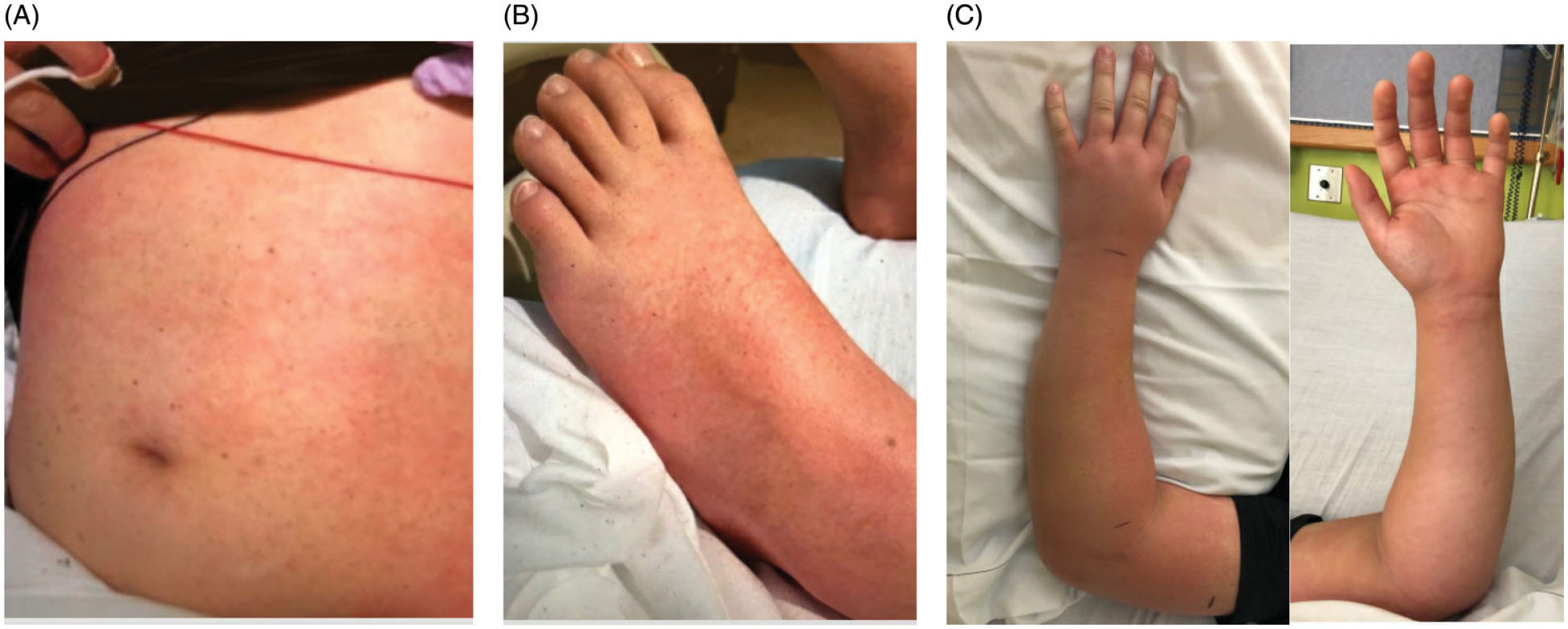
Cutaneous findings on day of presentation. A. Macular rash on trunk. B. Macular rash on lower extremities. C. The left forearm was moderately swollen and focally tender to the proximal ulna at the site of checking injury.

On presentation to the hospital, he was afebrile but vital signs were notable for tachycardia to the 110 s, hypotension in the 80/30 s, and O2 saturation of 95% on room air. Labwork on presentation is summarized in Table 1, most notable for elevated inflammatory markers and new-onset renal failure. He was found to have significant right lower quadrant tenderness and forearm edema without cellulitis, fluctuance, crepitus, or skin break. He was also noted to have a diffuse macular rash over the trunk, lower extremities, and axilla (Figure 1). Plain films and Doppler ultrasound of the forearm were normal. An ultrasound of the appendix was inconclusive and a follow-up CT abdomen revealed an 8 mm retrocecal mildly hyperemic appendix suspicious for early acute appendicitis. He was admitted to the pediatric ICU where he was started on IV ceftriaxone and metronidazole. Blood cultures and a respiratory panel were sent, as well as urinalysis given his acute kidney injury and hematuria.

**Table 1.** Laboratory results on day of admission.

Test	Results	Test	Results
WBC	7.7	ESR	70
Hemoglobin	11.4	CRP	>300
Hematocrit	33.9	Procalcitonin	33
Platelets	114	CK	422
		Lactate	2.2
Na	136		
Glucose	107		
BUN	42		
Creatinine	2.2	Respiratory Panel	Negative
Alkaline Phosphatase	87	Urinalysis	2+ blood, 2+ protein, nitrite +, leuk est +
ALT	29	Blood culture	*Streptococcus pyogenes*
AST	41		

**Figure 2.**
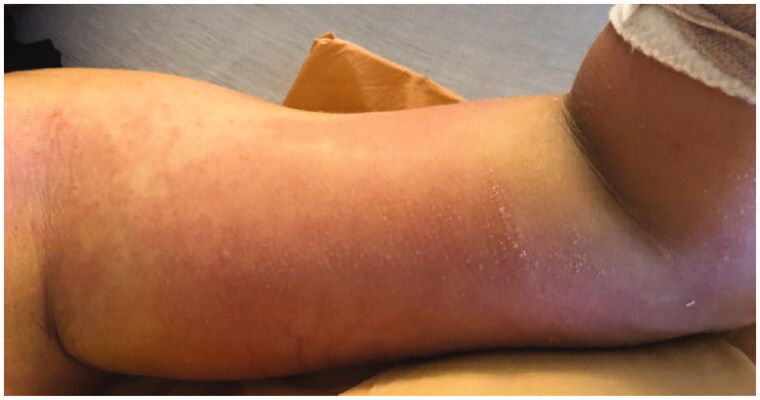
Morning of operative intervention; note new vesicle formation on medial upper arm and progression of macular rash.

Infectious disease and orthopedic surgery were consulted. His compilation of symptoms were concerning for toxic shock syndrome. LRINEC score was calculated to be 7 given the lab work summarized in Table 1. After discussion with the family regarding his forearm as a potential source of infection, he underwent an MRI with contrast which demonstrated diffuse subcutaneous edema in the forearm and bony edema in the proximal ulna, possibly consistent with his recent checking injury. Radiology felt there was no evidence of myositis or necrotizing fasciitis. After completion of 24 h of IV antibiotics, his abdominal complaints improved but he had minimal systemic improvement and increasing erythema of the arm as well as a new vesicular rash on the medial left arm (Figure 2). After discussion with the patient and his parents, he was taken emergently to the operating room for irrigation and debridement of the left forearm approximately 36 h after hospital presentation.

**Figure 3.**
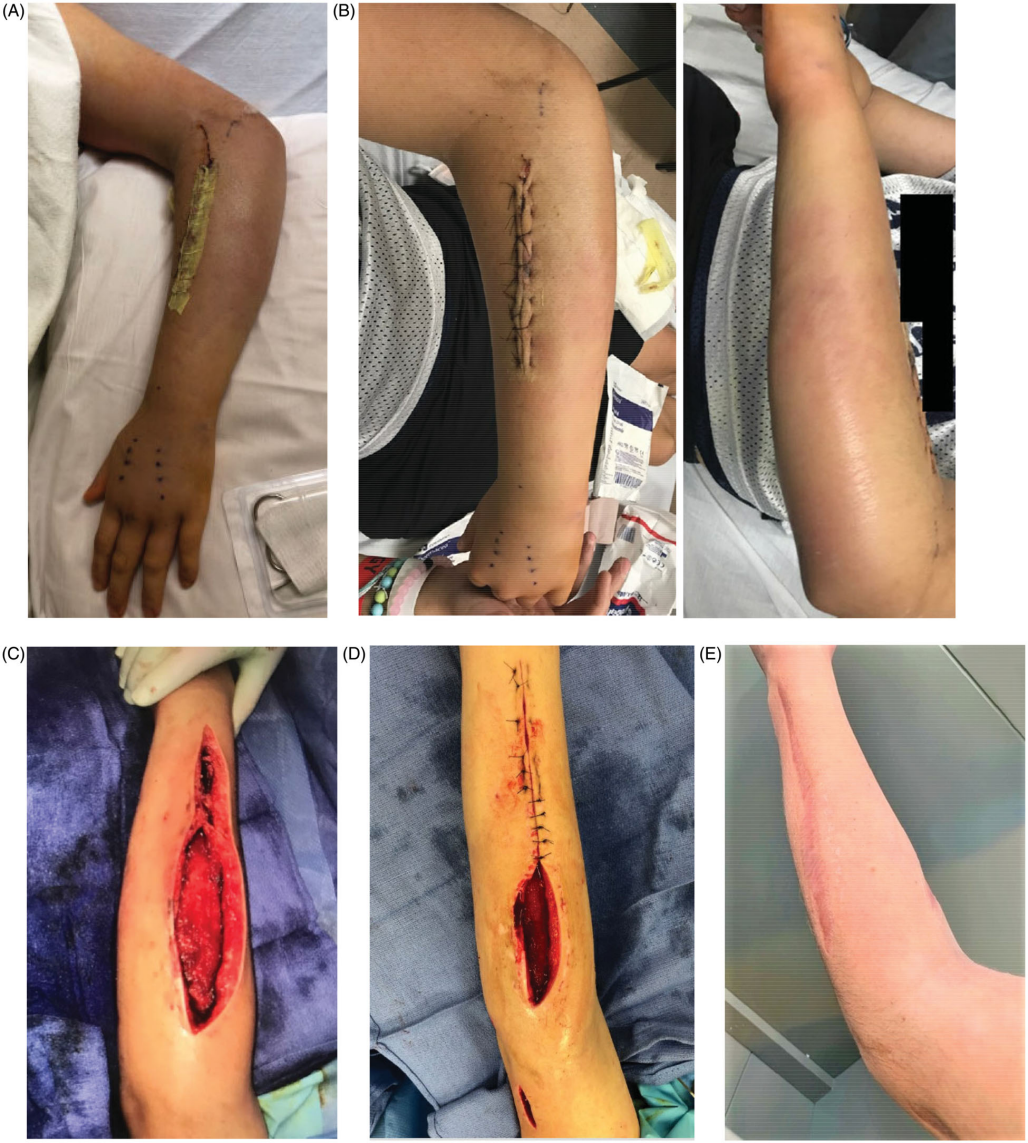
Appearance of forearm after (A) initial post-operative day two, with improvement in swelling and rash. (B) Readmission on post-operative day six with increased swelling. (C) Fourth operative irrigation and debridement. (D) Sixth and final irrigation and debridement with partial closure. (E) One year post-operatively.

During surgical debridement, murky dishwater fluid was encountered in the proximal forearm superficial to the fascia at the site of checking injury. This appeared localized and there was no evidence of fascial tracking or of muscle necrosis, with all muscle bellies examined and found to be healthy and viable. Thorough irrigation was performed with 9 L of normal saline and a forearm drain was placed prior to loose closure. Operative and blood cultures grew group A streptococcus and his antibiotics were transitioned from ceftriaxone to oxacillin and clindamycin after consultation with the infectious disease team. He experienced marked improvement in his symptoms. By post-operative day (POD) one, his rash and diarrhea resolved and creatinine and inflammatory markers downtrended. His drain was removed by POD 2 (Figure 3(A)). On POD 5, his CRP downtrended from >300 to 89 and he was deemed stable for discharge on a course of oral cephalexin as directed by the infectious disease team.

Two days after discharge he was readmitted for high fevers to 103 and emesis. His arm swelling had increased slightly since discharge (Figure 3(B)). Repeat MRI revealed reaccumulation of fluid in the proximal forearm. Repeat irrigation and debridement was performed with extension of the incisions. Murky fluid was again encountered at the proximal forearm though no fascial tracking was appreciated, and several drains were placed. Operative cultures from the second procedure were negative. The patient again demonstrated rapid clinical improvement. PICC line was placed and he was discharged on IV cefazolin and clindamycin three days after his second surgery with close infectious disease follow-up.

The following morning he presented to the clinic with increased pain. He was found to have new tenderness and swelling involving the elbow and dorsal wrist and increased CRP to 68 (from 34 two days prior). He was readmitted for more extensive irrigation and debridement involving the upper arm, elbow, and extending to the dorsal wrist and hand. Over the subsequent three weeks, a total of six operative irrigation and debridements were conducted. No muscle necrosis was noted during any operative intervention and the fascial integrity appeared maintained. Apart from the index procedure, all operative cultures were negative. Wounds were covered with wound vacuum-irrigator and eventually transitioned to damp-to-dry packing changes. After the final debridement, the forearm incision was closed primarily (Figure 3(C)) and the upper arm wound was left open to heal by secondary intention. By index POD 33 he had complete resolution of all symptoms, and normalization of inflammatory markers by index POD 45. His intravenous course of cefazolin/clindamycin was continued for four weeks, followed by an oral course of cefalexin and clindamycin for an additional two weeks. His proximal wound had completely healed by index POD 78. He worked extensively with occupational therapy and regained full elbow range of motion and was cleared to return to sports.

## Discussion

Streptococcal soft tissue infections and toxic shock syndrome (STSS) are serious and invasive infections that require prompt diagnosis and treatment. While necrotizing and non-necrotizing soft tissue infections from *Streptococcus pyogenes* are well-described following minor skin trauma, cryptogenic manifestations are also possible without any evidence of skin trauma. Our patient sustained a blunt checking injury without any cutaneous lesions, but developed a persistent deep infection which required multiple debridements. The etiology of cryptogenic infections is not well-known but is thought to relate to the seeding of the deep tissues after transient bacteremia from the nasopharynx. Interestingly, our patient’s younger brother had recently recovered from strep pharyngitis, and persistent pharyngeal carriage of group A strep in children is common even if they are asymptomatic [7]. In these patients with cryptogenic streptococcal infections and minimal cutaneous findings, crescendo pain is the most important diagnostic clue which precedes end-organ dysfunction [4]. A macular rash, vesicles or blistering may manifest, as in our patient, though this is relatively uncommon [8].

The typical treatment of necrotizing fasciitis includes serial debridement every 1-2 days until there is no residual necrotic tissue. After initial debridement, our decision to proceed with serial debridements for this patient despite his atypical course was based upon clinical examination and the patient’s escalating CRP values. A previous study of pediatric patients with NSTI suggests that CRP >20 is the most sensitive marker of NSTI [9], and our patient’s inflammatory markers were carefully trended and correlated closely with clinical recurrence. In retrospect, wounds may have been left open after initial debridement with a plan for scheduled takeback and delayed closure, which may have reduced the risk of recurrence.

Negative-pressure wound therapy (NPWT) helps facilitate the closure and healing of open wounds [10]. Although the use of NPWT is controversial in cases of NSTI, it is routinely used to treat necrotizing fasciitis of the head and neck in otolaryngology literature and has been shown to reduce the amount of excised skin in debridement and clear accumulating secretions from the wound [11]. Wound vac therapy is also effective in treating deep or ‘hidden’ space infections while minimizing exposure, and has also been found to improve the condition of post-debridement NSTI wounds. This may be related to its ability to prevent eschar development or pseudomembrane formation [12,13]. In this instance, we chose to use an alternating wound vacuum-irrigator to facilitate continued wound irrigation. Intravenous clindamycin in addition to penicillin is recommended for at least a 10–14 day course [4]; in this instance, the oral regimen on initial discharge was likely insufficient to treat this patient’s infection.

Although we did not identify any fascial tracking or muscle necrosis, and his MRIs were inconsistent with necrotizing fasciitis, this patient’s infection was unusually tenacious and required a lengthy operative course. The persistent nature of group A strep is due to a number of unique structural properties. Streptococcal superantigens, and in particular exotoxins such as the SpeK exotoxin, have been implicated in the invasive development of STSS [3,14]. Certain subsets such as M-type Streptococcus are also more likely to cause aggressive disease [15]. Prior case reports also suggest the role of a fascial biofilm in persistent group A Strep infections [16]. A multicenter study identified a biofilm in 32% of 31 patients for whom fascial, muscle, and soft tissue biopsy was obtained during repeat debridement for necrotizing soft tissue infection [16]. They concluded that characteristics associated with biofilm formation included higher bacterial load, septic shock, and pronounced inflammatory response *via* elevated IL-8 and neutrophilic predominance. Fiedler et al. have identified a number of structural properties and virulence factors, such as surface proteins, pili, secreted enzymes, and cytotoxic activity towards host cell types, which facilitate the formation and persistence of Strep biofilm [17,18]. Some studies have suggested that as many as 90% of group A Streptococcal serotypes have the ability to form biofilm *in vivo* [19,20].

The diagnosis of streptococcal toxic shock syndrome remains elusive. While early symptoms are often very nonspecific, the final stage often results in rapid decompensation and a high mortality rate [21].

Many of these systemic presentations are related to an underlying soft tissue infection. In the event of culture-isolated group A streptococcal infections and bacteremia, or in patients hospitalized for STSS, providers should maintain a high suspicion for indolent or cryptogenic soft tissue infection, even in the setting of a benign clinical appearance.
